# Variations in the Extensor Pollicis Brevis-Extensor Pollicis Longus Tendon Complex

**DOI:** 10.7759/cureus.52249

**Published:** 2024-01-14

**Authors:** Reiji Nishimura, Tohru Hashimoto, Tohru Yano, Hideaki Bo, Kazuhiro Maeda, Masataka Okabe, Takeshi Miyawaki

**Affiliations:** 1 Plastic and Reconstructive Surgery, The Jikei University School of Medicine, Tokyo, JPN; 2 Anatomy, The Jikei University School of Medicine, Tokyo, JPN; 3 Orthopedics, The Jikei University School of Medicine, Tokyo, JPN

**Keywords:** development, evolution, extensor pollicis longus, muscle-tendon module, anomaly, extensor pollicis brevis

## Abstract

Despite several reports on the running of the extensor pollicis brevis (EPB) tendons, the classification of tendon insertions remains ununified due to differences in reports. This diversity in tendon patterning is attributed to the process of tendon development. In this study, we assessed the running of the EPB tendons of 44 cadaver hands fixed in ethanol/formalin in detail and examined the existing classification method. The specimens were obtained from 15 women and seven men, with an average age of 86 years. Consistent with previous reports, we observed a wide diversity in the running of the EPB tendons. Further, we found that EPB tendon insertions showed diverse variations in the proportion and running of fibers, making it difficult to classify them into independent patterns. It is speculated that the EPB tendon develops through a different process than that of the muscle body of the EPB and that the entire muscle-tendon module of the EPB is evolving. The diversity of the EPB tendons observed in this study may reflect the ongoing process of evolution. In clinical practice, a wide variation in the running of the EPB tendons should be considered.

## Introduction

During extension of the thumb, the extensor pollicis longus (EPL), extensor pollicis brevis (EPB), and extensor hood all originate from the metacarpophalangeal (MP) to the interphalangeal (IP) joint, forming a complex [1-2]. Among the elements that make up this complex, the EPB is known to have a large number of variations [3-5]. Because the muscle bodies of the EPB and abductor pollicis longus (APL) are often continuous, the EPB has been considered a separate structure from the APL [6]. In addition, the diversity of the EPB, including its tendons, is believed to arise due to the evolution of the developmental process of the EPB muscle [7]. Conversely, recent studies have revealed that limb muscles and tendons have different ontogenetic processes [8-9]. Although there are many reports on the running of the EPB tendons, the classification of tendon insertions differs among reports and is not unified [1-3,5,10-16]. Thus, the present study aimed to observe the running and insertion of the EPB tendons in detail and examine their classification from the perspective of the process of tendon development.

## Materials and methods

We examined the running of the EPB, EPL, and APL tendons in 44 hands of 22 Japanese cadavers fixed in ethanol/formalin. The specimens were obtained from 15 (68%) women and seven (32%) men, with an average age of 86 years (69-105 years). The skin from the forearm to the hand was carefully removed, and all extensor tendons inserted into the thumb were dissected. The extensor retinaculum and tendon sheath covering the tendons were removed, and the running of the extensor tendons from the insertions to the myotendinous junction was analyzed. The muscular part of the extensors was not evaluated, as it was difficult to discern the boundaries of the adjacent muscle bellies in ethanol/formalin-fixed specimens.

In addition to macro-anatomical observations, we selected a few hands with an indistinct boundary at the confluence of the EPB and EPL tendons for histological examination, from which we excised the EPB-EPL complex portion. The excised specimens were stretched flat, embedded in resin, and sectioned at a thickness of 5 μm parallel to the fiber run. The sections were stained with toluidine blue, and the running of tendon fibers at the confluence of the EPB and EPL tendons was observed under the microscope. Based on the resulting gross and histological observations, we discussed the diversity, development, and clinical importance of the EPB tendons.

Ethical approval was obtained from the Institutional Review Board of the Jikei University School of Medicine, Tokyo, Japan (approval number: 32-425, approval date: 2022/7/4).

## Results

**Figure 1:**
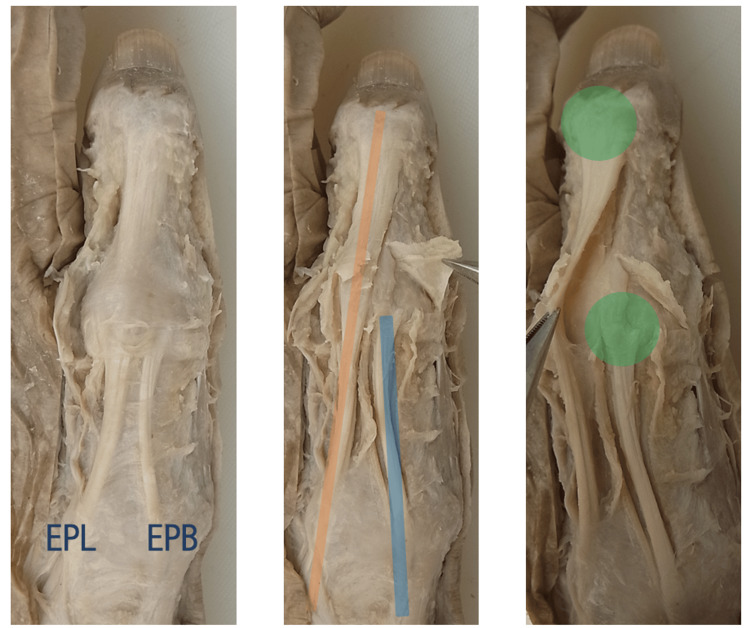
Standard running of the EPB and EPL tendons The left hand of the female cadaver. The EPL attaches to the distal phalanx base, while the EPB attaches to the proximal phalanx. No merging of tendon fibers is observed. EPB: extensor pollicis brevis; EPL: extensor pollicis longus

**Figure 2:**
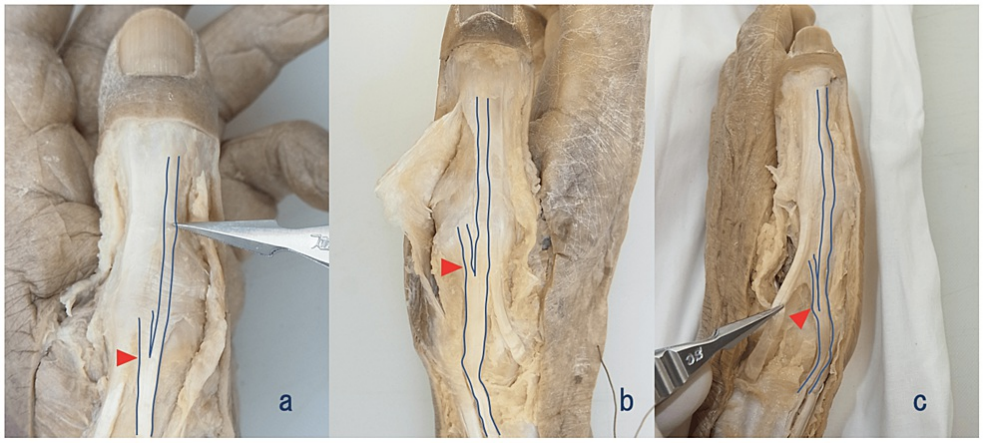
Diverse proportions of branching EPB tendon fibers In some cases, the branching EPB tendon was inserted into the distal IP and MP joints. We observed diverse proportions of branching tendon fibers, and at the MP joint level, they were inserted into the aponeurosis, or proximal phalanx base. (a) The left hand of the male cadaver. (b) The left hand of the male cadaver. (c) The left hand of the female cadaver. Arrowheads indicate branching sites of the EPB tendon. EPB: extensor pollicis brevis; MP: metacarpophalangeal; IP: interphalangeal

**Figure 3:**
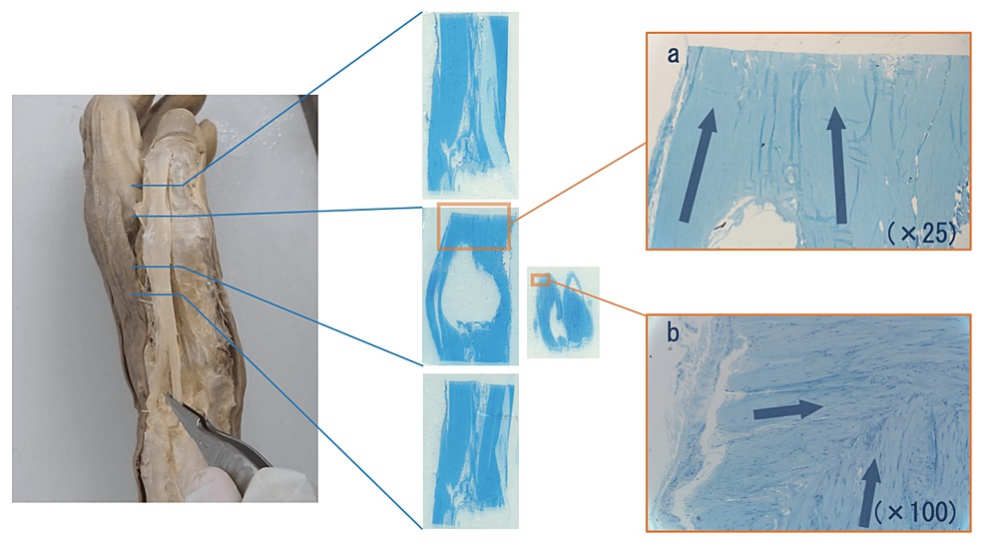
The direction of tendon fibers at the merging site of the EPB and EPL tendon The left hand of the male cadaver. The merging site of the EPB and EPL tendons was stained with toluidine blue to examine the direction of tendon fibers. (a) The fibers of the EPB and EPL tendons were merged at the distal MP joint. (b) At the tendon cap, fibers in the long-axis direction were merged with fibers in the short-axis direction in a weaving manner. EPB: extensor pollicis brevis; EPL: extensor pollicis longus; MP: metacarpophalangeal

**Figure 4:**
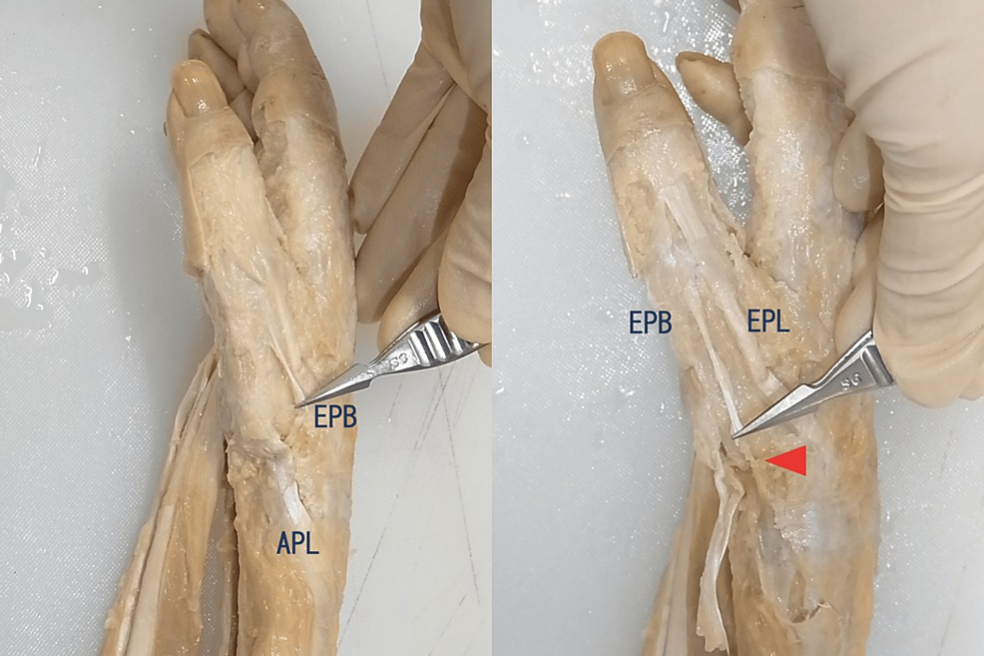
A case with a defect of the EPB tendon The right hand of the female cadaver. We found an accessory tendon with no muscles connecting the proximal phalanx base and the distal first extensor tendon compartment. The arrowhead indicates the origin of the accessory tendon. EPB: extensor pollicis brevis; EPL: extensor pollicis longus; APL: abductor pollicis longus

**Figure 5:**
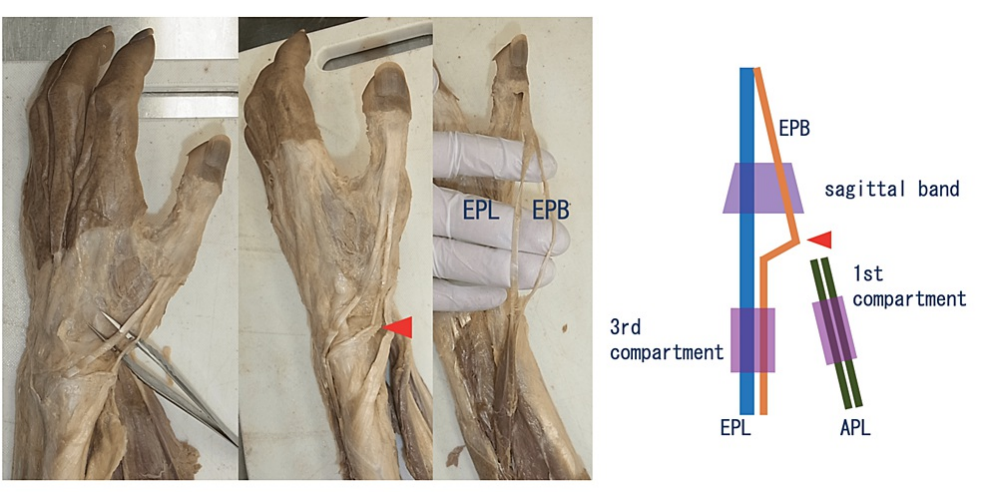
Variant course of the EPB tendon in the third extensor compartment The left hand of the male cadaver. The EPB tendon changed its direction at the distal first compartment (arrowhead) and ran through the third compartment. This EPB tendon was continuous with the EPL tendon at the proximal site. EPB: extensor pollicis brevis; EPL: extensor pollicis longus; APL: abductor pollicis longus

The EPL tendon attaches to the base of the distal phalanx, and the EPB tendon is inserted into the base of the proximal phalanx without exchanging fibers with the EPL tendon. This standard running of the tendons was observed in 24 (19 female and five male) out of 44 hands (Figure 1). At least part of the EPB tendon fibers reached the distal phalanx in 16 hands. In these hands, there were varying proportions of fiber bundles of the EPB tendons branching from the main trunk. In some cases, all the EPB tendon fibers reached the distal phalanx without branching, while only small fiber bundles branched and reached the distal phalanx in other cases. Moreover, the fiber bundles branching from the main trunk of the EPB tendons were not uniform as they were inserted into either the aponeurosis, proximal phalanx, or EPL tendon (Figure 2). These variations in EPB tendon fibers showed a continuous change from a state where they are independent of the EPL to a state where they are completely merged with the EPL rather than classifiable independent patterns. In a case with an unclear intersection boundary between the EPB and EPL tendons, histological observation of the running of tendon fibers showed that the fibers of the EPB and EPL tendons were completely merged at the level of the distal MP joint (Figure 3a). Furthermore, at the extensor hood, fibers in the long-axis direction were merged with fibers in the uniaxial direction in a weaving manner (Figure 3b). Regarding other variations, a defect of the EPB tendon was found in three hands, two of which had an accessory tendon between the base of the proximal phalanx and the distal part of the first compartment (Figure 4). On the one hand, the EPB tendon ran in the third compartment rather than the first, and the EPB tendon was continuous with the EPL tendon (Figure 5). There were no cases in which the EPB and APL tendons were continuous.

## Discussion

Diversity in the running of the EPB tendons

The diversity in the EPB is widely known; as Dawson stated, “The EPB has so many anatomical variations that deviations from the norm are not considered exceptions” [5]. The insertion sites of the EPB tendon reported thus far include the proximal phalanx, distal phalanx, hood, first metacarpal, or a combination thereof [1,2,5,10-16] (Table 1). In addition, variations other than the insertion sites include EPB defects and accessory/abnormal tendons. The following elements may explain why the classification of insertion sites differs among reports [1,2,5,10-16]: (1) The proportions of fibers branching around the MP and variations in the running are so diverse that intermediate types can exist for any pattern classification. The thin fibers branching from the main trunk of the EPB tendon around the MP joint intermingle with the fibers of the hood/EPL tendon, making identification of the insertion site difficult. When the EPB and EPL tendons are merged, the tendon fibers intermingle in a weaving manner without boundaries [17], as shown in this study. This is similar to a structure in which one tendon is divided into multiple branches [15]. Furthermore, in some cases, the EPB tendon fibers merge to the hood and extend continuously to the distal phalanx [2]. The EPB tendons are thus speculated to have a structure with various intermediate types, rather than a structure divided into independent patterns. (2) Variations in the EPB tendon may differ greatly depending on the population studied. A study conducted in Africa reported that the insertion of the EPB tendon into the proximal phalanx was observed in only 5% of cases, while at least some fibers reached the distal phalanx in most cases [17]. A report from India showed that insertion of the EPB tendon into the first metacarpal was observed in 3% of cases [15]. It is speculated that these variations in the EPB tendon among populations demonstrate instability in the process of evolution.

**Table 1:** Variations in the insertion of the EPB tendon The insertion sites of the EPB tendon are diverse, and their classification differs among reports. The EPB tendon is often inserted into multiple sites, making its classification difficult. EPB: extensor pollicis brevis

Author	Hands	① Proximal phalanx	② Distal phalanx	③ Hood	④ 1st metacarpal	Absent	①＋②	①＋③	②＋③	①＋②＋③	①＋④
Dawson and Barton [5]	16	25% (4)	0	0	0	19% (3)	0	56% (9)	0	0	0
Brunelli and Brunelli [10]	52	0	8% (4)	69% (36)	0	4% (2)	0	19% (10)	0	0	0
Kulshreshtha et al. [2]	44	25% (11)	0	2% (1)	0	0	0	25% (11)	0	0	0
Joshi et al. [11]	110	58% (64)	27% (30)	0	0	0	15% (16)	0	0	0	0
Alemohammad et al. [12]	90	79% (71)	0	4% (4)	0	0	0	0	0	17% (15)	0
Roy et al. [13]	98	81% (79)	14% (14)	0	0	0	5% (5)	0	0	0	0
Shigematsu et al. [14]	144	22% (32)	9% (13)	29% (42)	0	8% (11)	0	19% (28)	13% (18)	27% (12)	0
Ravi et al. [15]	77	57% (44)	35% (27)	0	3%(2)	1% (1)	3% (2)	0	0	0	1% (1)
Takahashi et al. [1]	100	34% (34)	20% (20)	46% (46)	0	0	0	0	0	0	0
Öztürk et al. [16]	83	16% (13)	0	17% (14)	0	0	0	57% (47)	8% (7)	2% (2)	0
Present study	44	55% (24)	2% (1)	0	0	7% (3)	37% (16)	0	0	0	0

In the present study, one hand showed the EPB tendon running through the third compartment, which had been previously reported in an autopsy case, and one case was discovered incidentally during surgery [18-19]. Although cases of abnormal EPL tendons are rare due to their stable structure in contrast to the EPB tendon, several cases of EPL tendon duplication have been reported [20]. Therefore, the EPB tendon running through the third compartment can be considered a duplication of the EPL tendon with a defect in the EPB tendon.

Association with clinical practice

EPB tendon variations are important for clinical practice. EPB variations may directly impact the development of de Quervain's disease by altering the mechanical conditions within the first extensor compartment of the wrist [3]. Interestingly, the incidence of distal EPB insertion on the distal phalanx is higher in patients with separate first compartments, suggesting a relationship between EPB variation and inter-tendinous septum [12]. In addition, EPB tendon variations may have a significant impact on trauma outcomes, diseases, or tendon transfer surgeries. Full extension of the thumb IP joint may be maintained by the EPB tendon even when the EPL tendon is completely ruptured; it is speculated that the symptoms produced by a rupture of the EPL tendon may vary according to EPB tendon variations [21]. Differences in insertion sites of the EPB tendon may affect the mechanical balance between the flexors and extensors of the thumb and thus may be involved in thumb deformities caused by rheumatoid arthritis [22]. Although several tendon transfer surgeries using the EPB tendon or its accessory tendon have been reported [4,23,24], the results of surgeries using the EPB tendon may be greatly influenced by EPB variations. Identifying the variations of the EPB tendon will help clinicians understand and treat problems in the hand.

Development of the EPB tendon

Because the EPB muscles are often fused with the APL muscles, it has been thought that the entire muscle-tendon module of the EPB had separated from the APL during evolution [25]. However, muscles and tendons are derived from different cells and have different developmental processes. In fact, unlike muscles, the EPB and APL tendons rarely merge, although they are positioned very close to each other [26]. On the other hand, it is not uncommon for a part of or the entire EPB tendon to merge with the EPL tendon [15,27].

Muscle progenitor cells in the upper limbs are derived from somites (paraxial mesoderm), while tendon progenitor cells are derived from the limb mesenchyme (lateral plate mesoderm) [28]. During muscle development, myoblasts first migrate to the limb bud, forming muscle masses on the dorsal and ventral sides. Division of the muscle masses results in the development of individual muscles. The muscle masses of extensor precursors are divided into three compartments, and each is then divided into individual muscles. The APL, EPL, and extensor indicis propius muscles develop from the same deep portion as the EPB muscle [7]. Among extensor precursors, the division of the deep portion is particularly unstable, suggesting that it may be undergoing evolution [7]. The hand (autopod) and forearm (zeugopod) undergo different mechanisms during tendon development. At the early stage of tendon patterning, tendons in the hand develop depending on cartilage, while tendons in the forearm develop depending on muscles [8-9]. Eventually, the tendon primordium developed in the wrist joint area connects the forearm muscles and the hand tendons, integrating them as a module from the forearm muscles to the insertion site of the hand tendons while interacting with muscles and cartilage [8]. The mechanically unnatural rerouting of the EPB tendon through the third compartment may have resulted from the tendon originating as an EPB tendon in the distal portion joining the muscle formed as an EPL in the proximal portion. Although the mechanisms that control individual tendon patterning remain unknown [29], the diversity of the EPB tendons suggests that tendon patterning is evolving, as with the division of muscles. The fact that variations in the insertion site of the EPB tendon are often not the same between the left and right sides [5,10,11] also supports the instability of the EPB tendon patterning.

EPB and evolution

Humans and other primates share a common muscle structure of the hand and forearm [6]. However, humans and hylobates are the only primates that have the EPB with independent muscle bodies [6]. The EPB of hylobates is inserted into the first metacarpal or carpal bone rather than the phalanges. Gorillas have a tendon similar to the EPB inserted into the proximal phalanx, but their muscle bodies are not separated from the APL. The ability to change the patterning of muscles and tendons is useful for environmental adaptation, as it is generally linked directly to function [28]. For this reason, it has been pointed out that a mechanism that integrates muscles and tendons with different developmental primordia into functional modules and partially changes each module without changing the basic design of development may have been beneficial to human evolution as a species [28,30]. In addition to the EPB, an independent flexor pollicis longus (FPL) that is not continuous with the flexor digitorum profundus is also a characteristic human structure, and this FPL and the EPB inserted into the proximal phalanx work simultaneously to allow independent flexion of the IP joint while keeping the MP joint in extension [6]. This posture may have been useful in the production and use of stone tools [6,29]. Because the function of the EPB is important for performing grasping actions and using tools, the muscle-tendon module involving the human EPB may still be in the process of evolution. It is, therefore, necessary to elucidate the mechanisms that control the tendon patterning in the hand to understand the reasons for the diversity of the EPB tendons and predict the direction of changes in a module.

Limitation

This study has several limitations that should be considered. Firstly, the limited number of cases in which the fiber pattern of the EPB-EPL complex could be observed in this study is an insufficient basis for forming definite conclusions. A comprehensive histological examination of tendon fiber runs may reveal whether the fiber distribution variations between EPL and EPB form a spectrum or have a non-random pattern.

In the present study, our investigation was limited to tendons. Investigation over the entire length of the muscle-tendon module may yield new insights into the development of the upper extremity extensor system through clarification of the relationship between tendon portion and muscle portion variation.

## Conclusions

While many variations of EPB tendons have been reported, their classifications have not been unified. We observed that EPB tendon fibers branch in varying proportions and stop at multiple locations or merge into the EPL tendon. This suggests that variations in EPB tendons may be an unclassifiable spectrum. It is possible that this diversity results from the fact that the EPB is a relatively new structure unique to humans and is still in the process of evolution. If so, the diversity of EPB tendons may be more than just an anomaly and may even be a major regional and ethnic variation. EPB tendon variations are clinically important because they are involved in the function of the thumb and may influence the development of de Quervain's disease and the outcomes of tendon transfer surgery. Clarification of EPB tendon fiber running without a specific classification may help clinicians gain a more accurate understanding of hand problems and better treatment options.
